# Genomic alterations in DNA repair and chromatin remodeling genes in estrogen receptor-positive metastatic breast cancer patients with exceptional responses to capecitabine

**DOI:** 10.1002/cam4.464

**Published:** 2015-04-13

**Authors:** Maren K Levin, Kai Wang, Roman Yelensky, Ying Cao, Corinne Ramos, Nicholas Hoke, John Pippen, Joanne L Blum, Barry Brooks, Gary Palmer, Norma Palma, Sohail Balasubramanian, Jeffrey S Ross, Joyce O’Shaughnessy

**Affiliations:** 1 Baylor-Sammons Cancer Center, Texas Oncology, US OncologyDallas, Texas; 2 Foundation Medicine Inc.Cambridge, Massachusetts; 3 Valley Medical Oncology ConsultantsPleasanton, California; 4 Theranostics Health Inc.Rockville, Maryland; 5 Albany Medical CollegeAlbany, New York

**Keywords:** capecitabine, metastatic breast cancer, exceptional responders, DNA damage response, chromatin remodeling genes

## Abstract

We analyzed the genomic and phosphoproteomic profiles of breast cancer tissue obtained from six patients with estrogen receptor (ER)-positive, HER2-negative metastatic breast cancer who had highly durable (≥5 years) and, in some cases, ongoing clinical responses with capecitabine. Formalin-fixed, paraffin-embedded tissue samples from patients’ primary (*n* = 4) or metastatic (*n* = 2) breast cancers were utilized for targeted next-generation sequencing and reversed phase protein microarray. Two patients received capecitabine monotherapy. Four patients received capecitabine in combination with paclitaxel; three of these continued single-agent capecitabine after stopping paclitaxel. Capecitabine was discontinued for progressive disease after a mean of 66 months in four patients (range 54–86 months), and two patients remain on therapy, having received capecitabine for >91 months and >122 months, respectively. Three patients’ cancers (50%) had likely functional alterations in DNA repair and chromatin remodeling genes, while three other patients’ cancers had variants of unknown significance in these pathways. Mutations in *PIK3CA*, amplifications of *FGFR1* or *ZNF703*, or phosphorylation of HER family receptors and their downstream proteins did not preclude exceptional responses to capecitabine. None of the patients’ tumors harbored *TP53* or *PTEN* mutations. Four of the patients had breast cancer tissue available for PTEN immunohistochemistry, and all four patients’ cancers were positive for PTEN. These surprising findings in a group of phenotypically similar patients with ER-positive, endocrine therapy-pretreated, HER2-negative metastases, are supported by preclinical data showing that sensitivity to 5-fluorouracil is enhanced by deficiencies in chromatin remodeling and homologous recombination genes. Our findings suggest that mutations that inactivate homologous recombination and/or chromatin remodeling genes within ER-positive, HER2-negative breast cancers may predict for highly durable responses to capecitabine.

## Introduction

There is considerable interest in oncology in prospectively identifying genomic alterations that may predict for response to a given therapy, and in selecting patients for standard or investigational therapies based on predefined genetic alterations. There is also an emerging interest in identifying patients who have had an exceptional response to a therapy, and in evaluating these patients’ cancers for molecular alterations that may account for the observed marked benefit 1. Such information could prove valuable in informing future treatment recommendations for phenotypically similar patients whose cancers harbor the same molecular alterations.

Capecitabine has proven efficacy as monotherapy for the treatment of patients with locally advanced or metastatic breast cancer (MBC) after failure of anthracycline- and taxane-containing chemotherapy, or in patients for whom further anthracycline therapy is not indicated. Capecitabine has been used successfully in the treatment of MBC since 1998 2, thus creating the opportunity to identify and investigate the characteristics of exceptional responders in the clinic. However, very long durations of response to capecitabine are uncommon. Here, we describe the clinical phenotype, cancer genotype, and phosphoproteomic profiles for six MBC patients whose metastatic disease responded to capecitabine for at least 5 years.

## Methods

Patients with MBC who had received capecitabine monotherapy or capecitabine in combination with a taxane followed by single-agent capecitabine over the course of at least 5 years were identified from two practices in Texas Oncology. Institutional Review Board-approved informed consent for tissue collection and molecular analysis was obtained in accordance with Baylor University Medical Center requirements.

Formalin-fixed, paraffin-embedded (FFPE) tissue samples were prepared from primary breast cancers (*n* = 4) or metastatic disease (*n* = 2) from patients with ≥5 years of response to capecitabine as treatment for metastatic disease. Targeted next-generation sequencing (NGS) was carried out at a Clinical Laboratory Improvement Amendments (CLIA)-certified laboratory (Foundation Medicine Inc., Cambridge, MA) on at least 50 ng of extracted DNA using the Illumina HiSeq at an average depth exceeding 500× in order to characterize genomic alterations across 287 cancer-related genes. An evaluation of all classes of genomic alterations was performed, including base substitutions, short insertions/deletions, focal amplifications, homozygous deletions, and gene fusions/rearrangements, as previously described 3. In order to maximize detection accuracy (sensitivity and specificity) in commonly impure clinical specimens, the test was optimized and validated to detect genomic alterations with high accuracy 4.

Phosphoprotein analysis was also performed on the FFPE samples using a reversed phase protein microarray (RPMA) platform at a CLIA-certified laboratory (Theranostics Health, Inc., Rockville, MD). Immunostaining was carried out with 14 antibodies directed against specific phosphorylated, cleaved, or total proteins within the HER family (HER1, p-HER1 [Tyr 1068], HER2, p-HER2 (Tyr 1248), HER3, p-HER3 (Tyr 1289), p-Akt (Ser 473), p-mTOR (Ser 2448), p-S6 ribosomal protein (Ser 235–236), p-4E-BP1 (Ser 65), p-MEK1/2 (Ser 217–221), p-ERK1/2 (Thr 202-Tyr 204), p-Jak2 (Tyr 1007–1008), and p-STAT3 [Tyr 705]), in order to characterize the activity of downstream signaling pathways known to be involved in breast cancer pathogenesis. The scoring of each analyte was determined by comparing the normalized fluorescence intensity per unit of protein (NFU) value in the patient sample of interest with that of a representative population. The scores assigned corresponded to the number of standard deviations (SD) from the population mean for each analyte.

Phosphatase and tensin homolog (PTEN) immunohistochemistry (IHC; monoclonal antibody, clone 6H2.1) with 0+ or ≤50% positive cells was regarded as negative for PTEN staining, and ≥1+ and >50% positive cells was regarded as positive. Androgen receptor (AR) IHC (monoclonal antibody AR318) with <10% positive nuclei was regarded as negative for AR staining.

## Results

### Clinical phenotype

Six postmenopausal patients with MBC who had had exceptional clinical responses to capecitabine, and for whom adequate FFPE archival tissue for molecular analysis existed, were identified (Table S1). Evaluation of the patients’ primary breast cancers, as well as metastatic samples where available, revealed that they were all estrogen receptor (ER) positive and HER2 negative. Five of the six patients (83.3%) had received prior anthracycline and/or taxane therapy, and all six patients (100%) had been previously treated with endocrine therapy before beginning capecitabine. Four patients (66.7%) had received chemotherapy for metastatic disease prior to initiating capecitabine. Initially, all patients received capecitabine twice daily at the standard dosing schedule of 14 days on, 7 days off (see Table S1 for capecitabine doses). Four patients received capecitabine in combination with paclitaxel for a mean of 17 months (range 4–57 months); three of these patients continued with capecitabine monotherapy for a mean of 86 months (range 59–118 months) after discontinuing paclitaxel for toxicity, and the fourth patient continued with combined capecitabine plus paclitaxel. Two patients received capecitabine as a single agent (54 months and 91+ months, respectively). Five of the patients had liver metastases, two of whom also had bone metastases, and one patient had liver, bone, and chest wall involvement. The sixth patient had bone predominant disease. Capecitabine was discontinued after a mean of 66 months in four patients (range 54–86 months), but two patients remain on capecitabine therapy alone, having received treatment for >91 months and >122 months, respectively.

### Genotype analyses

Cancers from three patients (50%; patients 2, 3, and 4) had likely functional alterations in DNA-damage-response and chromatin remodeling genes (Table1), which is higher than the published prevalence in ER-positive breast cancer 6. Three patients’ cancers (50%; patients 1, 5, and 6) had variants of unknown significance (VUS) in DNA repair and chromatin remodeling genes, namely base substitutions, INDELs (insertions or the deletion of multiple bases), or truncations, of unknown functional significance in breast cancer 2, early onset (*BRCA2*), Fanconi anemia complementation group (*FANCF*), SET domain containing 2 (*SETD2*), poly (ADP-ribose) polymerase 1 (*PARP1*), nuclear receptor corepressor 1 (*NCOR1*), core-binding factor subunit b (*CBFB*), and E1A binding protein 300 (*EP300*). Mutations in the phosphoinositide-3-kinase, catalytic, alpha polypeptide gene (*PIK3CA*), or amplifications of fibroblast growth factor receptor 1 (*FGFR1*) or zinc finger protein 703 (*ZNF703*) genes, did not preclude the development of a highly durable response to capecitabine (Table1). None of the patients’ tumors carried tumor suppressor 53 (*TP53*) mutations or homozygous *PTEN* deletions on NGS.

**Table 1:** Genomic alterations identified via NGS

Patient	Genomic alterations or *[VUS]*			
	DNA repair	Chromatin remodeling	PI3K pathway	Other genomic alterations
1	[*BRCA2*, *FANCF*]	[*SETD2*]	*PIK3CA* mutation	*MCL1* amplification
2	*CHEK2*, *PALB2*	*NCOR1*, *TET2*	–	*RB1* deletion
3	*CHEK2*	[*BCORL1*]	–	–
4	*ATM*	*EP300*	*FGFR1* amplification	*MYC*, *ZNF703* amplifications
5	[*PARP1*]	[*NCOR1*, *CBFB*]	*PIK3CA* mutation	*CDH1*, *GATA3* mutations
6	[*SETD2*] 5	[*EP300*, *SETD2*]	–	–

Results in brackets represent variants of unknown significance (VUS). The VUS are short variants defined as base substitutions, INDELs (insertions or the deletion of multiple bases) or truncations, of unknown functional significance.

### Phosphoproteomic analysis

Evidence of phosphorylation of HER family receptors and their downstream signaling proteins in the patients’ cancers on RPMA analyses was found to not preclude having a highly durable response to capecitabine (Table2). Activation of HER1, HER2, and HER3 as well as PI3K and signal transducer and activator of transcription 3 (STAT3) pathways was observed in both the primary and even more so in the MBC samples analyzed (Table2).

**Table 2:**
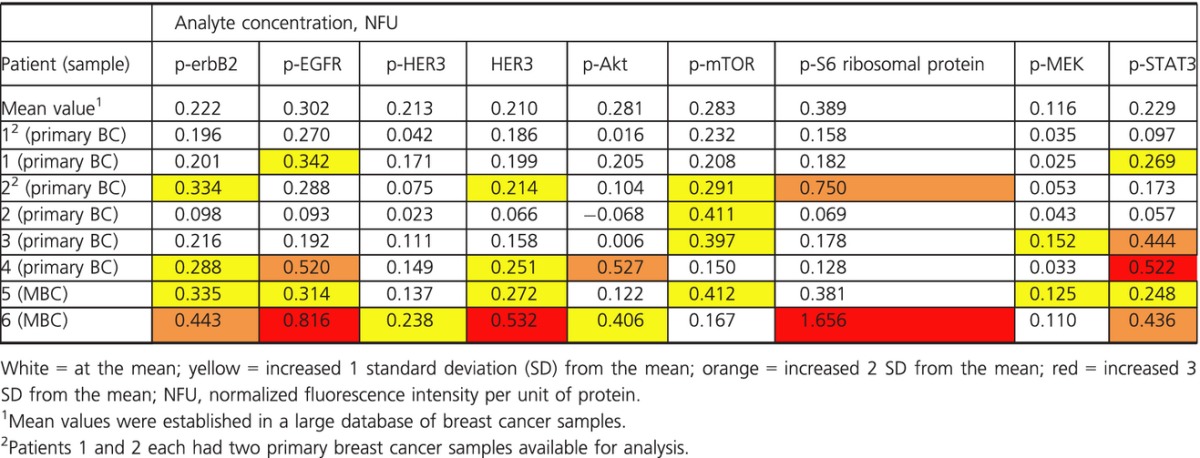
Phosphoproteome results

Four of the patients had breast cancer tissue available for PTEN IHC, and all four patients’ cancers were positive for PTEN (data not shown). AR expression was negative in the four patients (patients 1, 2, 3, and 5) who had sufficient tissue available for analysis (data not shown).

## Discussion

Median progression-free survival times for patients with anthracycline- and taxane-pretreated MBC who received single-agent capecitabine in randomized phase II/III clinical trials ranged from 3.1 to 6.6 months 7–12. Here, we describe six patients with exceptional responses to capecitabine (duration ranging from 54 to >122 months), three of whom had cancers with genomic alterations that likely led to dysfunctional DNA-damage-response (checkpoint and homologous recombination), and chromatin remodeling genes. We are currently exploring the potential function of the DNA damage response and chromatin remodeling VUS observed in the other three patients’ cancers. These surprising findings of very highly durable benefit from capecitabine in a group of phenotypically similar patients with ER-positive, endocrine therapy-pretreated, HER2-negative MBC, are supported by preclinical data showing that sensitivity to 5-fluorouracil (5-FU) is enhanced by deficiencies in chromatin remodeling and/or homologous recombination genes 13. 5-FU induces double-strand breaks in DNA, which are repaired by homologous recombination or postreplication repair (PRR) 14. Deficiency in chromatin modifier function decreases chromatin relaxation, preventing access of homologous recombination and PRR proteins to the DNA, thereby interfering with double-strand DNA damage repair. Genomic alterations that decrease the effectiveness of these two DNA repair pathways simultaneously lead to a greater impact on the cell cycle and G2/M replication arrest with 5-FU than do deficits in only one DNA repair pathway 13. We hypothesize that the chromatin remodeling and homologous recombination defects observed in our patients’ cancers prevented repair of 5-FU-induced DNA damage by interfering with two key DNA repair pathways (homologous recombination and PRR), leading to marked replication arrest. It is not known whether a single genomic alteration in either a chromatin remodeling or a homologous recombination gene would enhance clinical sensitivity to capecitabine.

Genomic alterations in the PI3K pathway were identified in three of the patients’ cancers, demonstrating that these presumed activating alterations do not preclude prolonged benefit from capecitabine. HER family pathway activation (p-erbB2, p-EGFR, p-HER3) was common in the primary and MBC samples, with downstream activation of p-mTOR and p-STAT3 seen in several patients’ cancers. Four of the patients’ cancers with sufficient tissue available for analysis were positive for PTEN on IHC. This observation raises the question of whether intact PTEN is a requirement for exceptional response to capecitabine. Interestingly, this observed activation of HER family signaling also did not preclude the development of highly durable responses to capecitabine.

We queried The Cancer Genome Atlas (TCGA) ER-positive/HER2-negative breast invasive carcinoma cases 15 using the cBioPortal 16,17 and found that the DNA repair and chromatin remodeling gene alterations identified in our patients’ cancers occurred in 1% to 4% and in 0% to 5% of TCGA cases, respectively. We did not find evidence of co-occurrence of the DNA repair and chromatin remodeling genomic alterations as observed in the capecitabine exceptional responders in the TCGA ER-positive, HER2-negative primary breast cancers, suggesting that coexpression of these alterations is uncommon.

We analyzed primary or MBC tissues obtained from nine patients who had ER-positive, HER2-negative MBC and whose disease had responded to capecitabine for less than 2 years. None of these patients’ cancers had alterations in chromatin remodeling genes, and only one of the nine cancers contained a somatic mutation in a DNA-damage-response pathway gene, *BRCA2* (data not shown).

The interplay between AR and ER expression in breast cancer is under investigation. Four of the patients’ cancers that we were able to examine had no AR staining on IHC. This finding is in contrast to data from a large cohort of breast cancer patients (*n* = 5521), which revealed the presence of AR expression in approximately 80% of ER-positive cancers on expert central pathology evaluation 18. Whether the absence of AR in these ER-positive cancers is an essential component of the molecular profiles that characterize these exceptional responders is unknown.

In summary, molecular analyses of cancer tissue obtained from six ER-positive, HER2-negative MBC patients who had exceptional responses to capecitabine suggest that functional alterations in DNA-damage-response and chromatin remodeling genes may predict for prolonged response to capecitabine within this phenotypic context. This report is unusual in reporting six exceptional responders’ common phenotype and genotype, as most reports have described fewer patients with exceptional response 1,19. It would be of interest to corroborate these findings by prospectively identifying ER-positive, HER2-negative MBC patients who have liver and/or bone metastases and whose cancers harbor the described genotype, to evaluate their duration of response to capecitabine.

## Supporting Information

**Table S1.** Clinical histories of the six patients with exceptional responses to capecitabine.
