# Supplementary material for: Genomic alterations in DNA repair and chromatin remodeling genes in estrogen receptor-positive metastatic breast cancer patients with exceptional responses to capecitabine
Source: Cancer Med. 2015 Apr 13;4(8):1289–93. doi: 10.1002/cam4.464 (PMC4559040; doi:10.1002/cam4.464)
Supplement: Supplementary file 1 [file cam40004-1289-sd1.doc]

**Supporting information**

**Table S1.** Clinical histories of the six patients with exceptional responses to capecitabine

| **Patient; age***  **Breast cancer diagnosis** | **ER/PgR/HER2 status** | **Prior A/T therapy** | **Site(s) of metastatic disease** | **Chemotherapy for metastatic disease** | **Capecitabine dosing schedule** | **Outcome on capecitabine** | **Total time on capecitabine** |
| --- | --- | --- | --- | --- | --- | --- | --- |
| **1**  44 years  2002: bilateral ductal/lobular breast cancer with diffuse lytic bone metastases | +/+/-  (primary sample; MBC sample not analyzed) | None | 2002: bone  2003: liver | Mar 2003: XP started | XP x10:  X 2,500ª2,000 mg 14/7 + weekly P 80 mg/m^2^ d1, d8  Oct 2003: complete response in liver and bone; P stopped due to grade 3 HFS; X continued alone 2,500 or 3,000 mg 14/7 | Feb 2007: PD in bone, new sacral pain, rising CA27.29, no liver disease; letrozole added  Sep 2008: PD in bones on PET/CT scan; X/letrozole discontinued | 66 months (5.5 years); 7 months XP, 59 months X |
| **2**  47 years  1998: left clinical inflammatory breast cancer | +/+/-  (primary and MBC sample) | 1998: neoadjuvant AC; mastectomy; adjuvant T x4 ªHDC CTCb/ABMT; radiation and tamoxifen | 2000: liver | Jan 2002: XP started | XP: X 3,500 mg 14/7 + P 80 mg/m^2^, 3 weeks on, 1 week off  Apr 2002: P dose changed to 80 mg/m^2^,  2 weeks on, 1 week off  Jul 2002: Diffusely fibrotic liver on CT scan; X continued alone (slowly decreasing dose to 2,500 mg 14/7 due to diarrhea and HFS) | Apr 2008: PD in liver; peritoneal lymph nodes positive; X discontinued | 86 months (7.2 years); 5 months XP, 81 months X |
| **3**  38 years  1987: right breast lumpectomy, T2N0ª breast radiotherapy; no adjuvant treatment  1993: recurrence right breast, grade 3, underwent mastectomy | +/+/-  (primary and MBC sample) | 1993: AC x6 🠪 tamoxifen for 1.5 years | 1998: chest wall  2000: bone | 1998: P x3, stopped due to neuropathy  1998: HDC CTCb/ABMT, locoregional radiation and tamoxifen  2000: P + exemestane x6  2002: XP started | XP: X 1,500 mg bid 14/7  + weekly P 80 mg/m^2^ d1, d8 with excellent response  P stopped due to severe HFS and nail toxicity | 2004: stable dose of X 1,000 mg bid 7/4; grade 1–2 HFS and fatigue, bone scans stable and abdominal adenopathy resolved  Slow normalization of serum CEA and bone scan stable with only slight activity at T12  Jan 2011: X continues; no evidence of PD on 2,500 mg 7/7 | 122 months (10.2 years) to date; 4 months XP, X treatment continues |
| **4**  56 years  2002: right breast invasive ductal carcinoma | +/+/-  (primary and MBC sample) | 2002: mastectomy; adjuvant AC x4 ªtamoxifen | 2006: liver  2011: liver and bone | 2006: Cb + T  2007: Bev added; complete response, Bev discontinued  2007: X alone started | X: 3,500 mg 14/7  2009: X dose schedule changed to 7/7 | 2011: PET scan showed metastases in liver, left scapula and T7 vertebral body; liver metastases proven by biopsy; X discontinued | 54 months (4.5 years) X |
| **5**  33 years  1976: left breast mastectomy | +/-/-  (MBC sample) | 1980: oophorectomy ªprolonged CMF treatment | 1980: chest wall  2004: bone, liver, and chest wall | 2004: exemestane, pamidronate, and radiation to C-spine  2004: AC x3 ªP x3 ªCb + P with partial response  2005: X alone started | X 2,500 mgª2,000 mg ª1,500mg 14/7 due to HFS | Jun 2005: CT scan showed improvement in liver disease  X continues; dermal nodules and liver lesions disappeared | 91 months (7.5 years) to date; X treatment continues |
| **6**  35 years  1998: left upper outer quadrant multifocal invasive ductal carcinoma | +/-/-  (MBC sample) | 1998: bilateral mastectomies; AC x4 ªP x2; switched to T x2 due to poor toleranceª tamoxifen for 5 years | 2004: bone 2006: liver | 2004: letrozole + pamidronate; letrozole switched to exemestane then to fulvestrant  Feb 2006: irinotecan + Cb  Apr 2006: liver metastases; XP started | XP:  X 2,500 mg 14/7ª1,000 mg bid + weekly P 100 mg/m^2^ d1, d8ª80 mg/m^2^ after cycle 1 due to mild neutropenia  X dose schedule changed to 12/7 by cycle 7 for improved tolerability | 2008: resolution of liver metastases; bone metastases remained stable by imaging studies, asymptomatic without bone pain  Jan 2011: bone metastatic progressing; XP discontinued | 57 months (4.8 years) XP |

*Signifies age at diagnosis.

ABMT = autologous bone marrow transplant; AC = doxorubicin and cyclophoshamide; A/T = anthracycline/taxane; Bev = bevacizumab; Cb = carboplatin; CEA = carcinoembryonic antigen; CMF = cyclophosphamide, methotrexate, and fluorouracil; CT = computerized tomography; d = day; ER = estrogen receptor; HDC CTCb = high-dose chemotherapy of cyclophosphamide, thiotepa and carboplatin; HFS = hand-foot syndrome; MBC = metastatic breast cancer; P = paclitaxel; PD = progressive disease; PET = positron emission tomography; PgR = progesterone receptor; T = docetaxel; X = capecitabine.
